# South-Western Michigan Dental Association

**Published:** 1897-07

**Authors:** F. H. Essig


### South-western Michigan Dental Association.

The meeting of the South-western Michigan Dental Association was held in Kalamazoo, April 13th and 14th, 1897, in the Academy of Medicine rooms in the Public Library building and was called to order by the president, Tuesday, at 2:30 P. M. An address of welcome was delivered by Dr. Rockwell of Kalamazoo, secretary of the Academy of Medicine, which was well received. The president's semi-annual address consisted of advice to the younger members of the profession. A committee of three was appointed by the Chair to draft resolutions on the death of Dr. Ray of St. Joseph, Mich. The following were appointed: Drs. Rockwell, White and Backus; Dr. Backus, chairman.

The first paper—"How can we best educate the masses in dentistry," by Dr. S. M. Fowler, of Muskegon, Michigan. The paper was well received and called forth a lively discussion. The discussion was opened by Dr. R. M. Speer, of Battle Creek, Mich., followed by remarks from Dr. Abell, Albion, Mich.; Dr. Rockwell, Kalamazoo, Mich.; Dr. Rockwell, Benton Harbor, Mich.; Dr. J. Taft, University of Michigan; Dr. C. R. Rowley, Chicago and Dr. Siddell, Kalamazoo. Paper, "Judgment," by Dr. Blackmarr, of Jackson, Mich. Discussion opened by Dr. C. F. Siddell, of Kalamazoo, Mich., and remarks were made by Drs. Taft, Rockwell and J. Ward House, of Grand Rapids. This closed the papers for the afternoon. Dr. Condit of Chicago demonstrated his removable bridge, which was well received. The meeting then adjourned until 7:30 P. M..

At 7:30 P. M. the meeting was again called to order by the president. A paper was read by Dr. S. M. White of Benton Harbor, Mich., subject, "How can we increase the interest in our Dental Societies?" Discussion opened by Dr. A. C. Runyan of South Haven, Mich. Remarks were made by Drs. C. J. Siddell, Abell, Taft, Rix and Rowley. A motion was then made and carried, to adjourn to attend the banquet at the American House at 9 P. M. and to meet again Wednesday morning at 9 A. M.

The meeting was again called to order Wednesday morning at 9 A. M. Paper, subject: "Pyrozone, its uses in dentistry," by Dr. E. A. Honey, of Kalamazoo, Mich. Discussion opened by Dr. Rowley of Chicago. Remarks by Drs. Siddell, Runyan, Blackmarr and Abell. Paper by Dr. H. C. Rockwell, of Benton Harbor, Mich.: "Value of Experience in Dentistry. Remarks were make by Drs. H. C. Runyan, House and Siddell, also Drs. Blackmarr, Rowley Diack and Taft. Paper by Dr. M. P. Green, Kalamazoo. Subject: "Pulp Mumification." Discussion opened by Dr. Codding. Remarks by Drs. Rockwell, Essig and Abell. Paper read on " Biography of Dr. H. W. Ray. Moved and supported that a committee be appointed to look after the death of members, prominent dentists, and prepare a memorial to be presented at each meeting. The president appointed Dr. Blackmarr, Dr. Rockwell and Dr. Honey: A vote was taken on new members; vote carried unanimous.

Wednesday, P. M., meeting was called to order by the president at 2:30 Business of Society: Having received an invitation from Benton Harbor, Mich., to hold the next regular meeting there. It was accepted to be held there the second Tuesday and Wednesday in September. A motion was made and carried that this society extend an invitation to the Northern Indiana Dental Association to attend the Fall meeting at Benton Harbor. The president then appointed Drs. Rockwell, Jarvis, Backus, White and Elsworth as the executive committee. On motion the dentists of Kalamazoo were tendered a vote of thanks for the excellent hospitality shown the society during the meeting. On motion the Academy of Medicine was tendered a vote of thanks for the use of their beautiful rooms. On motion Dr. Rockwell, of Kalamazoo, was tendered a vote of thanks for his able address of welcome. The meeting then adjourned until the second Tuesday in September, 1897- F. H. Essig, D.D.S., *Secretary.*

